# DON in pediatric cerebral malaria, a phase I/IIA dose-escalation safety study: study protocol for a clinical trial 

**DOI:** 10.1186/s13063-023-07808-w

**Published:** 2024-01-26

**Authors:** Nginache Nampota-Nkomba, Osward M. Nyirenda, Jane Mallewa, Yamikani Chimalizeni, Nettie Dzabala, Michael P. Fay, Mathangi Gopalakrishnan, Matthew B. Laurens, Nicole F. O’Brien, Louis H. Miller, Susan K. Pierce, Brittany A. Riggle, Douglas G. Postels

**Affiliations:** 1 Blantyre Malaria Project, Kamuzu University of Health Sciences, Blantyre, Malawi; 2 Department of Internal Medicine, Kamuzu University of Health Sciences, Blantyre, Malawi; 3 Department of Paediatrics and Child Health, Kamuzu University of Health Sciences, Blantyre, Malawi; 4 Department of Pharmacy, Kamuzu University of Health Sciences, Blantyre, Malawi; 5 Biostatistics Research Branch, National Institute of Allergy and Infectious Diseases, National Institutes of Health, Rockville, MD USA; 6 Center for Translational Medicine, University of Maryland School of Pharmacy, Baltimore, MD USA; 7 Center for Vaccine Development and Global Health, University of Maryland School of Medicine, Baltimore, MD USA; 8 Department of Pediatrics, Division of Critical Care Medicine, Nationwide Children’s Hospital, Columbus, OH USA; 9 Laboratory of Malaria and Vector Research, National Institute of Allergy and Infectious Diseases, National Institutes of Health, Rockville, MD USA; 10 Laboratory of Immunogenetics, National Institute of Allergy and Infectious Diseases, National Institutes of Health, Rockville, MD USA; 11 Division of Neurology, The George Washington University/ Children’s National Medical Center, Washington, DC USA

**Keywords:** 6-Diazo-5-oxo-L-norleucine, DON, Glutamine antagonist, Cerebral malaria, Malaria, *Plasmodium falciparum*, Africa

## Abstract

**Background:**

Despite treatment with highly effective antimalarial drugs, malaria annually claims the lives of over half a million children under 5-years of age in sub-Saharan Africa. Cerebral malaria (CM), defined as *Plasmodium falciparum* infection with coma, is the severe malaria syndrome with the highest mortality. Studies in the CM mouse model suggest that a T cell-mediated response underlies CM pathology, opening a new target for therapy in humans. This trial aims to establish the preliminary safety of one such novel therapy, the glutamine antagonist 6-diazo-5-oxo-L-norleucine (DON).

**Methods:**

In this phase I/IIa dose-escalation clinical trial, a single dose of intravenous (IV) DON is administered to three participants groups—healthy adults and adults with uncomplicated malaria, then pediatric participants with CM—to primarily assess safety. The secondary objective of this trial is to assess pharmacokinetics of DON over a range of doses. The open-label adult portion of the trial enrolls 40 healthy adults concurrently with 40 adults with uncomplicated malaria. Cohorts of 10 participants receive a single IV dose of DON with doses escalating between cohorts from 0.1 mg/kg, 1.0 mg/kg, 5.0 mg/kg, to 10 mg/kg. Following subsequent safety review, a randomized, double-blind, and placebo-controlled pediatric study enrolls 72 participants aged 6 months to 14 years with CM. The pediatric portion of the study minimally spans three malaria seasons including a planned interim analysis after 50% of pediatric enrollments. The first half of pediatric participants receive DON 0.1 mg/kg, 1.0 mg/kg, or placebo. Dosing for the second half of pediatric participants is informed by the safety and preliminary efficacy results of those previously enrolled. The pediatric portion of the study has an exploratory outcome evaluating the preliminary efficacy of DON. Efficacy is assessed by diagnostics predictive of CM outcome: electroencephalography (EEG), magnetic resonance imaging (MRI), and transcranial doppler (TCD), measured before and after DON administration. All participants with malaria receive standard of care antimalarials in accordance with local guidelines, regardless of study drug dose group.

**Discussion:**

This preliminary safety and efficacy study evaluates DON, a candidate adjunctive therapy for pediatric CM. If results support DON preliminary safety and efficacy, follow-up phase II and III clinical trials will be indicated.

**Trial registration:**

This trial was registered on ClinicalTrials.gov on 28 July 2022 (NCT05478720).

## Administrative information

Note: the numbers in curly brackets in this protocol refer to SPIRIT checklist item numbers. The order of the items has been modified to group similar items (see http://www.equator-network.org/reporting-guidelines/spirit-2013-statement-defining-standard-protocol-items-for-clinical-trials/).

**Table:** 

Title {1}	DON in pediatric cerebral malaria: A Phase I/IIa dose-escalation safety study.
Trial registration {2a and 2b}.	This trial was registered on Clinicaltrials.gov on 28 July 2022 – https://clinicaltrials.gov/ct2/show/NCT05478720. All items from the World Health Organization Trial Registration Data set were included.
Protocol version {3}	Current: Version 2.1 dated 04 August 2023. Adult Enrollment: Version 1.7 dated 16 October 2022
Funding {4}	The trial is funded by a grant from the Division of Microbiology and Infectious Diseases (DMID), National Institute of Allergy and Infectious Diseases (NIAID), U.S. National Institutes of Health (NIH)– Grant Number U01AI155300, Protocol Number 21–0005, Funding Mechanism PAR-18–633. BAR, LHM, SKP receive support from the Intramural Research Program of the NIH.
Author details {5a}	Nginache Nampota-Nkomba^1^, Osward M. Nyirenda^1^, Jane Mallewa^2^, Yamikani Chimalizeni^3^, Nettie Dzabala^4^, Michael P. Fay^5^, Mathangi Gopalakrishnan^6^, Matthew B. Laurens^7^, Nicole F. O’Brien^1,8^, Louis H. Miller^9^, Susan K. Pierce^10^, Brittany A. Riggle^10^, Douglas G. Postels^1,11^. 1. Blantyre Malaria Project, Kamuzu University of Health Sciences, Blantyre, Malawi. 2. Department of Internal Medicine, Kamuzu University of Health Sciences, Blantyre, Malawi. 3. Department of Paediatrics and Child Health, Kamuzu University of Health Sciences, Blantyre, Malawi. 4. Department of Pharmacy, Kamuzu University of Health Sciences, Blantyre, Malawi. 5. Biostatistics Research Branch, National Institute of Allergy and Infectious Diseases, National Institutes of Health, Rockville, MD, USA 6. Center for Translational Medicine, University of Maryland School of Pharmacy, Baltimore, MD, USA. 7. Center for Vaccine Development and Global Health, University of Maryland School of Medicine, Baltimore, MD, USA 8. Department of Pediatrics, Division of Critical Care Medicine, Nationwide Children’s Hospital, The Ohio State University, Columbus, OH, USA. 9. Laboratory of Malaria and Vector Research, National Institute of Allergy and Infectious Diseases, National Institutes of Health, Rockville, MD, USA. 10. Laboratory of Immunogenetics, National Institute of Allergy and Infectious Diseases, National Institutes of Health, Rockville, MD, USA. 11. Division of Neurology, George Washington University/ Children’s National Hospital, Washington, DC, USA.
Name and contact information for the trial sponsor {5b}	Sponsor: National Institute of Allergy and Infectious Diseases, 5601 Fishers Lane, Rockville, MD, US. Email: ocpostoffice@niaid.nih.gov Phone: 866–284-4107
Role of sponsor {5c}	This is an investigator initiated clinical trial. The funders played no role in the study design, collection, management, analysis, data interpretation, manuscript writing, or the decision to submit for publication.

## Introduction

### Background and rationale {6a}

Cerebral malaria (CM), defined as an otherwise unexplained coma in someone with *Plasmodium falciparum (Pf)* parasitemia, is the most lethal form of severe malaria [1, 2]. Ninety percent of CM mortality occurs in children, the vast majority of whom are less than 5 years old [3]. Despite treatment with the highly effective anti-malarial, intravenous (IV) artesunate, mortality rates are 15–25%, and children who survive often suffer severe neurological sequalae. At present, there are no predictive diagnostics for which children with malaria will develop CM nor therapy for CM after children progress to severe disease. Thus, the public health burden is enormous.

Current knowledge of the cellular and molecular mechanisms that underlie CM disease pathology are incomplete. The observation of heavily infected red blood cell (iRBC) sequestration in the cerebral vasculature of children who died of CM, often accompanied by intra- and peri-vascular pathology including ring hemorrhages, led to the generally accepted hypothesis that iRBC sequestration in the cerebral vasculature and the resulting mechanical obstruction lead to inflammation, impaired vasoregulation, and blood brain barrier (BBB) dysfunction thus causing this severe progression of malaria [4]. Therapies targeting the purposed downstream effects of sequestration were investigated in multiple clinical trials [5–19], but none improved clinical outcomes. These failures suggested that the downstream effects of iRBC sequestration were poor targets of therapies.

Studies in the mouse model of CM (referred to as experimental cerebral malaria or ECM) provided strong evidence for the role of parasite specific CD8^+^ T cells in the pathology of CM [20]. CD8^+^ T cells had not been targeted for CM therapy in humans, in part, because they had not been reported in the cerebral vasculature of children who died of CM [21, 22]. We recently provided definitive evidence for the presence of CD8^+^ T cells lining the cerebral vasculature in children who died from CM [23]. Our findings point to a mechanism by which CD8^+^ T cells function in disease pathophysiology based on the extraordinary similarities in the distribution of CD8^+^ T cells in the brains of children with CM and in mice with ECM. These observations open new avenues for treatment of CM that involve modulating CD8^+^ T cells with the wealth of available T cell targeting therapeutics.

This clinical trial aims to establish the preliminary safety and efficacy of one such novel therapy, 6-diazo-5-oxo-L-norleucine (DON), a glutamine antagonist. DON has a potent inhibitory effect on a variety of glutamine utilizing pathways including those involved in T cell metabolism. We showed that DON is highly efficacious in treating ECM in mice late in the disease when it has progressed to a point where significant brain swelling and BBB dysfunction have occurred [24]. DON both blocks the progression of ECM and promotes recovery [24, 25]. In a separate study, we used magnetic resonance imaging (MRI) to longitudinally monitor responses to treatment in mice with ECM. We found disease progression mirrored that observed in MRI studies in children with CM, and that, in mice, DON treatment resolved severe brain swelling, which is highly predictive of a fatal outcome [25, 26].

This is not the first clinical trial of DON in humans. DON safety in adults and children with cancer was established in phase I and II clinical trials performed over 40 years ago but did not enter therapeutic use due to a lack of consistent anti-tumor efficacy [17, 27–40]. Most of these studies were multi-dose, covered a wide range of DON treatment regimens, and all were in patients who had failed primary anti-cancer treatment courses. The most commonly reported adverse effects in these studies were nausea and vomiting. However, DON was not evaluated in a single dose, dose-escalation study with concomitant pharmacokinetic (PK) analysis. Therefore, before proceeding to efficacy trials of DON for treatment pediatric CM, we first evaluate DON’s safety in healthy Malawian adults and Malawian adults with uncomplicated malaria before moving to the target population, critically ill children with CM. In the dose-escalation phase I/IIa trial described herein, participants are premedicated with an antiemetic to prophylactically target the most common adverse effects. We will determine the safety profile and PK of a single dose of DON in healthy adult participants and adults with uncomplicated malaria over a dose range of 0.1 mg/kg to 10 mg/kg before assessing safety and efficacy in children with CM.

### Objectives {7}

The primary objective of this study is to evaluate the safety of a single IV dose of DON in healthy adults, adults with uncomplicated malaria, and children 6 months to 14 years old with World Health Organization (WHO) defined CM. Our secondary objective is to determine the PK profile of a single dose of DON administered to an individual at one of four doses studied. Our exploratory objectives are to both determine the preliminary efficacy of DON and to explore the metabolic mechanism(s) of action of DON in children with CM. We hypothesize that a single dose of DON will be safe and tolerable in healthy adults, adults with uncomplicated malaria, and children with CM over the studied dose ranges. We evaluate increasing doses of DON using preliminary markers of efficacy in children with CM, namely changes in patterns on continuous electroencephalography (cEEG), improved MRI brain volume scores, and improved flow velocities on transcranial doppler (TCD) all of which have been previously established as prognostic biomarkers associated with CM mortality [26, 41, 42].

### Trial design {8}

**Fig. 1:**
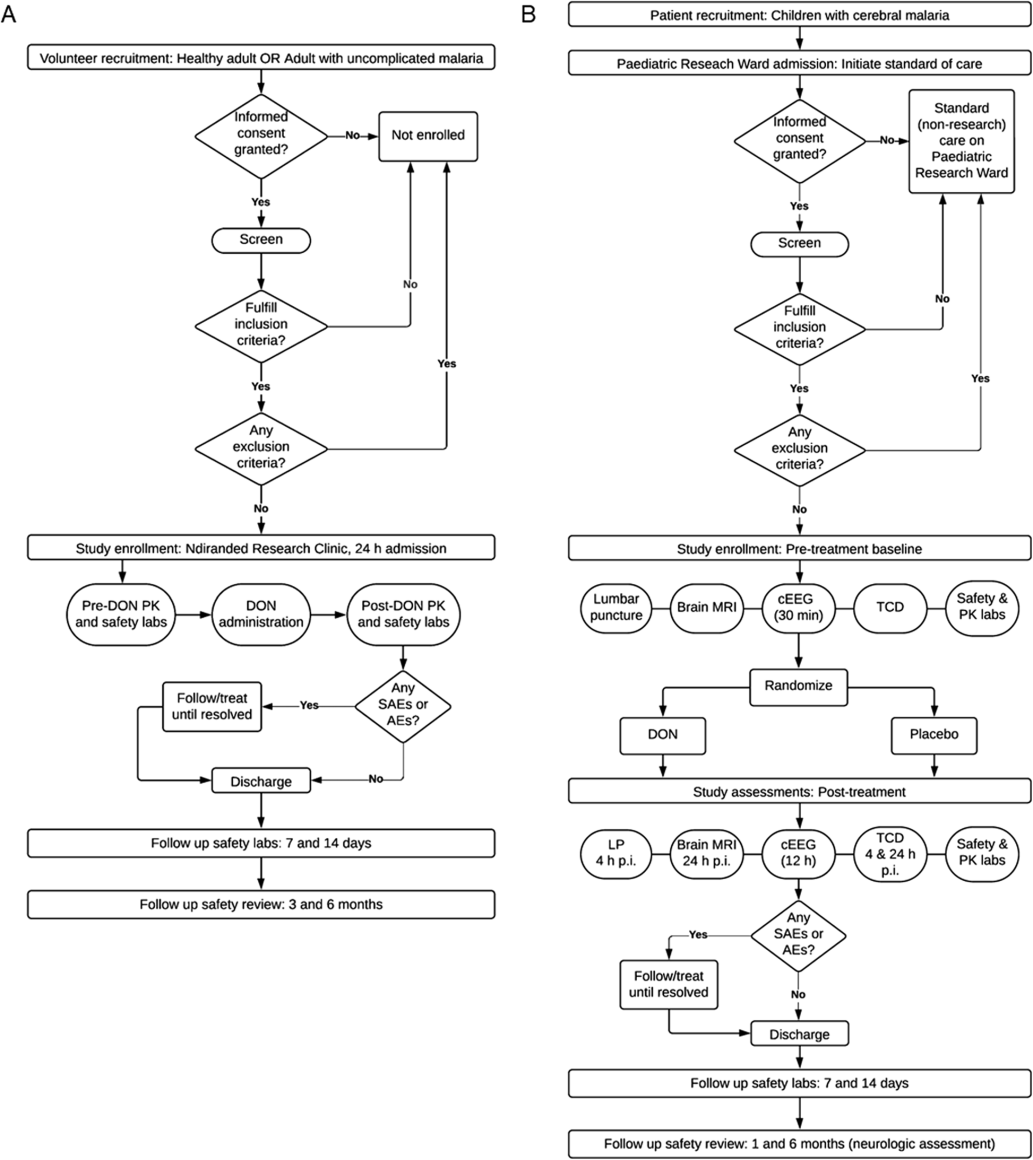
Schematic of study design for the adult (**A**) and pediatric (**B**) enrollment. Pharmacokinetic (PK); severe adverse events (SAEs); adverse events (AEs); continuous electroencephalogram (cEEG); magnetic resonance imaging (MRI); transcranial doppler (TCD); lumbar puncture (LP); post-intervention (p.i.)

This is a phase I/IIa dose-escalation clinical trial. DON is tested first in adults and then in pediatric participants to evaluate safety. In all participants, after baseline assessments and safety laboratory studies are complete, a single IV dose of DON is administered. The open-label adult portion of the trial concurrently enrolls 40 healthy adults and 40 adults with uncomplicated malaria. Within each of the two adult participant groups, cohorts of 10 participants receive a single IV dose of DON with dose escalation after study team safety review, from 0.1 mg/kg, 1.0 mg/kg, 5.0 mg/kg, to 10 mg/kg. The subsequent study in children with CM is randomized, double-blinded, and placebo-controlled. DON or placebo is administered to participants who also receive standard-of-care CM treatments including IV artesunate. Only doses of DON with a favorable safety profile in adults may be administered to pediatric participants. Four pediatric cohorts (cohorts 1–4) are enrolled, comprising up to 72 participants with CM aged 6 months to 14 years. Peak transmission occurs during the rainy season in Malawi, which is typically January–June. Anticipated enrollment spans at least three malaria seasons. Pediatric participants in cohorts 1 (*N* = 6, 8%) and 2 (*N* = 12, 17%) receive DON 0.1 mg/kg or placebo. Pediatric cohort 3 (*N* = 18, 25%) receives DON 1.0 mg/kg or placebo. Upon completion of cohorts 1–3, 50% of pediatric enrollment, there is an interim safety analysis. The remaining 50% of pediatric participants are enrolled in pediatric cohort 4. Dosing in cohort 4 is informed by both the adult safety data and the interim analysis of pediatric safety and exploratory efficacy data. Dosing may remain at the two lower doses of DON (0.1 mg/kg and 1.0 mg/kg) or may escalate to higher doses pending safety and preliminary efficacy results in children previously enrolled and regulatory review amendment approval. The duration of study for each participant is six months (Fig. 1).

## Methods: participants, interventions and outcomes

### Study setting {9}

This study is conducted in Blantyre, Malawi. Adults are enrolled at the Ndirande Research Clinic. Pediatric enrollments will occur on the Pediatric Research Ward (PRW) at Queen Elizabeth Central Hospital (QECH), the main referral hospital in the Southern Region of Malawi.

### Eligibility criteria {10}

Healthy adults are eligible for enrollment if they are not acutely ill and do not meet any exclusion criteria, including pregnancy, lactation, or current highly active antiretroviral (HAART) or anti-tuberculosis medication use (Table 1). Adults with uncomplicated malaria are enrolled if they have been diagnosed with malaria in the last 24 h, are febrile or have a history of fever, have a positive malaria thick blood smear, have a normal mental status, and are not taking any of the excluded medications mentioned previously. Children with CM are considered for enrollment if they have WHO defined CM: coma (defined as a Blantyre coma score (BCS) ≤ 2), a positive rapid diagnostic test or peripheral blood smear positive for malaria, and no other known causes of coma (Table 1). In all participants, screening and eligibility evaluations will be completed in less than 6 h.

**Table 1:** Inclusion and exclusion criteria for healthy adults, adults with uncomplicated malaria and children with cerebral malaria

	**Healthy adults**	**Adults with uncomplicated malaria**	**Children with cerebral malaria**
**Inclusion criteria**	Age ≥ 18 years old	Age ≥ 18 years old	Age 6 months–14 years old
	Informed consent obtained and informed consent form (ICF) signed	Informed consent obtained and ICF signed	Informed consent obtained and ICF signed by parent or guardian
	Temperature ≤ 37.5 °C	Temperature ≥ 38 °C or history of fever in the past 24 h	Temperature ≥ 38 °C or history of fever in the last 24 h
	Hemoglobin ≥ 7 g/dL or hematocrit/ packed-cell volume (PCV) ≥ 20%	Hemoglobin ≥ 7 g/dL or hematocrit/PCV ≥ 20%	Hematocrit or PCV ≥ 18%
	Thick or thin blood smear **negative** for asexual forms of *P. falciparum*	Thick or thin blood smear **positive** for asexual forms of *P. falciparum* (parasite count and speciation documented)	Thick or thin blood smear **positive** for asexual forms of *P. falciparum* or positive malaria rapid diagnostic test
	Negative pregnancy test for persons of child-bearing potential (9–59 years)	Negative pregnancy test for persons of child-bearing potential (9–59 years)	Negative pregnancy test for persons of child-bearing potential (≥ 9 years)
	Body mass index (BMI) 18.5–30 kg/m^2^	BMI 18.5–30 kg/m^2^	Blantyre coma score ≤ 2
	Creatinine: ≤ 110 mmol/L / ≤ 1.2 mg/dL (males) or ≤ 90 mmol/L / ≤ 1.0 mg/dL (females)	Creatinine: ≤ 110 mmol/L / ≤ 1.2 mg/dL (males) or ≤ 90 mmol/L / ≤ 1.0 mg/dL (females)	No other explanation for coma by history or physical exam
		Glasgow coma score of 15	
		Respiratory rate ≤ 20 breaths/minute	
		Oxygen saturation ≥ 90% on room air	
**Exclusion criteria**	Pregnancy or lactation	Pregnancy or lactation	Pregnancy or lactation
	Allergy to ondansetron	Allergy to ondansetron	Allergy to ondansetron or ceftriaxone
	Currently taking highly active antiretroviral therapy (HAART)	Currently taking HAART	Currently taking HAART
	Currently taking anti-tuberculosis medications	Currently taking anti-tuberculosis medications	Currently taking anti-tuberculosis medications
	Participants attempting to become pregnant	Participants attempting to become pregnant	Cloudy cerebrospinal fluid (probable bacterial central nervous system infection)
			Malnutrition > 3 standard deviations below the mean weight for height and/ or mid-upper arm circumference ≤ 11.5 cm

### Who will take informed consent? {26a}

Study nurses pre-screen potential study participants using an eligibility checklist. Adults or parents/guardians of pediatric potential participants are given a brief description of the study and, if interested in enrolling, are referred to either the Ndirande Research Clinic (adults) or PRW (children) for informed consent. Study nurses read the informed consent form (ICF) together with the potential adult participants or parents/guardians of potential pediatric participants. Key information about the study’s purpose, procedures and experimental aspects, risks and discomforts, expected benefits to the participant or to their parents/guardians, and alternative treatments (in pediatric participants, no adjunctive therapy) are reviewed. Adult participants and parents/guardians of pediatric participants sign the ICF before initiating study procedures. Minors will not be approached for assent as they are comatose and gravely ill at recruitment.

### Additional consent provisions for collection and use of participant data and biological specimens {26b}

The ICF includes information on possible analysis of participant biological specimens. Adult participants and parents/guardians of child participants will decide if they want any residual biological specimens destroyed or stored for possible future research at the end of the trial. The decision to participate in the storage of residual biological specimens can be changed at any time by notifying the study team.

## Interventions

### Explanation for the choice of comparators {6b}

The adult portion of the study does not have a comparator group. In contrast, the pediatric portion of the trial is placebo controlled to provide a comparator for exploratory efficacy endpoints. Placebo is normal saline in syringes identical to those used to deliver DON. Placebo is used because no approved treatment for CM yet exists.

### Intervention description {11a}

Individually packaged 200 mg or 10 mg fill vials of DON are stored frozen at –20 °C. Before administration, DON is removed from the freezer and allowed to reach room temperature over 30 min. Thawed DON is then reconstituted with 10 mL of normal saline. The reconstituted product is shaken to resolubilize, and a weight-specific volume of the active product is drawn up into an appropriately sized syringe. Thirty minutes after premedication with the antiemetic ondansetron, the intervention is infused IV over 10 min.

Within each of the two adult participant groups, healthy and uncomplicated malaria, the first 10 participants enrolled receive DON 0.1 mg/kg IV. If this dose is proven safe and tolerable, the subsequent cohorts of 10 participants in each adult group receive DON 1.0 mg/kg IV, 5.0 mg/kg IV, or 10.0 mg/kg IV, sequentially. In pediatric participants, we administer a single IV dose of DON with interim safety reviews. In cohort 1, we enroll 6 participants, randomized 2:1 to either DON 0.1 mg/kg or placebo. If no halting criteria (as described the “Criteria for discontinuing or modifying allocated interventions {11b}” section) are met, we continue to cohort 2 of pediatric recruitments in which 12 participants will be randomized 5:1 to receive DON 0.1 mg/kg or placebo, respectively (randomization ratio for DON 0.1 mg/kg or placebo of 7:2 cohort when combining cohorts 1 and 2). If the risk–benefit profile is promising, the study continues to cohort 3 of pediatric recruitment. In cohort 3, 18 pediatric participants receive either DON 1.0 mg/kg or placebo at a ratio of 7:2, respectively. Subsequent dose escalation above 1.0 mg/kg in pediatric participants are informed by safety and tolerability in the adult cohorts and in the first three pediatric cohorts and will not proceed without regulatory review. The doses in pediatric cohort 4 (*N* = 36) participants may either remain the same (DON 0.1 mg/kg, and 1.0 mg/kg), include one of the same doses, and/or include higher doses against placebo at an anticipated randomization ratio of 7:7:4 (DON dose 1: DON dose 2: placebo) subject to regulatory review and approval.

### Criteria for discontinuing or modifying allocated interventions {11b}

#### Adults

Dose-escalation in cohorts of adult participants halts if more than four of the 10 subjects in a dosing cohort have related grade 2 and 3 adverse events (AEs) or severe adverse events (SAEs), if there are related SAEs within any dose, or if there is an overall pattern of symptomatic, clinical, or laboratory events that the Pharmacovigilance Group within the Division of Microbiology and Infectious Diseases (DMID) or the Data and Safety Monitoring Board (DSMB) considers associated with the study product. The study will stop enrollment and describe findings to the independent safety monitors (ISM) and the DMSB to evaluate future dose escalation. At the end of adult enrollments, all safety data are reported to the DSMB to inform dose ranges to be studied in children with CM. The DMID medical monitor may stop enrollment and/or administration of study product if AEs meeting the halting criteria are reported.

#### Pediatric

We begin cohort 1 with 6 pediatric participants who receive the lowest dose of DON 0.1 mg/kg (*N* = 4) or placebo (*N* = 2). Barring significant SAEs triggering halting rules, we proceed to cohort 2 and enroll the remainder of the participants in the DON 0.1 mg/kg dose group with participants again randomized to DON 0.1 mg/kg (*N* = 10) or placebo (*N* = 2). If the number and type of SAEs incurred in cohort 1 and 2 do not meet study halting rules, we dose escalate in cohort 3 to DON 1.0 mg/kg (*N* = 14) or placebo (*N* = 4). After cohorts 1–3 of pediatric recruitment, an interim analysis will assess all safety and efficacy endpoints. If the benefit-risk profile is promising, the study continues to cohort 4 of pediatric recruitments. Based on the safety, pharmacokinetic, and preliminary efficacy profiles of the doses administered in cohorts 1–3, the dose(s) for participants in cohort 4 are determined after the interim analysis. The DSMB will meet after the completion of cohort 3 (50% of pediatric enrollment), and as needed for any study safety issue.

#### Pediatric halting rules

The pediatric study pauses enrollment if the number of participants with SAEs in any single MedDRA group exceeds rates expected in children with CM. The expected rates were derived from calculating the cumulative probability of stopping when the true rate of the proportion of participants in a specific MedDRA classification group is a given percent (i.e., 25, 30, 40 or 50%). For example, 9 participants out of the first 17 enrolled must have an SAE within a single MedDRA classification group for the study to pause for review. (This will indicate a single important toxicity that should be considered before further enrollments.) A halt is not triggered, however, if of the first 17 enrolled pediatric participants, 9 experience SAEs in different MedDRA classification groups (e.g., 3 participants die, 3 participants have renal failure, and 3 participants have severe thrombocytopenia). High rates of both adverse outcomes and abnormal laboratory values are expected in pediatric CM. Therefore, only single recurring SAEs within a MedDRA classification group will initiate a pause for safety review. The properties for the halting rules were estimated by Monte Carlo simulation with 100,000 replicates. At any halt, the DSMB will review the data and evaluate whether the study should stop due to safety concerns.

Although we will only report SAEs to the regulatory bodies that have a reasonable possibility of being related to the intervention, for the purpose of applying halting rules, all events (deaths, disability, etc.), even if not specifically reported as SAEs related to treatment, will be documented.

### Strategies to improve adherence to interventions {11c}

Participants are directly observed at the time of dosing by clinical research team members. Administration is documented on paper case report forms (CRFs) and entered into the electronic database. All study concomitant medications are administered under direct observation during the first 24 h after DON administration. Concomitant medication taken at home for up to 14 days after DON administration is followed until completion of the dosing schedule, and the stop date will be recorded in CRFs by study staff.

### Relevant concomitant care permitted or prohibited during the trial {11d}

#### Adults

Adult participants receive a premedication dose of the antiemetic ondansetron, 8.0 mg IV, administered 30 min before DON, and repeated 6 h later. Adult participants with uncomplicated malaria receive six doses (twice daily for 3 days) of oral Co-Artem® (a fixed dose combination tablet containing artemether, 20 mg and lumefantrine, 120 mg) for treatment of uncomplicated malaria, per Malawi Ministry of Health guidelines. All adult participants are infused with IV normal saline at 125 mL/h (maintenance) during the 24-h admission. Due to the possible teratogenic effects of DON on fetal development, all adult participants receive male condoms and are advised to use them for a minimum of 2 weeks following DON treatment.

#### Pediatric

Pediatric participants receive a premedication dose of ondansetron 0.15 mg/kg (maximum 5.0 mg) IV, administered 30 min before DON and repeated at 8 and 16 h after DON or placebo treatment. While on the clinical unit, all pediatric participants receive standard of care antimalarial treatment. IV artesunate is administered according to the current national treatment guidelines upon admission, at 12 and 24 h post-admission, and once a day thereafter until the child is able to take oral medications or tolerate nasogastric antimalarial administration, or for a total of 5 days, whichever comes first. After the child is able to take medications enterally, they are prescribed Co-Artem® (a fixed dose combination tablet containing artemether, 20 mg and lumefantrine, 120 mg) twice daily for 3 days.

All pediatric patients require a nasogastric tube and feeds are held for the first 24 h post-DON administration to decrease the risk of aspiration while comatose. Other supportive treatment for all participants is provided as clinically indicated.

### Provisions for post-trial care {30}

Should study participants become ill during trial follow-up and present to the study site, immediate medical treatment is provided by the study team and referred for appropriate health care if needed. Clinical trial insurance was obtained for all participants enrolled in this trial. Participants are informed of the existence of the insurance.

### Outcomes {12}

Primary and secondary endpoints in adult and pediatric cohorts assess safety and tolerability. As the risk–benefit profile and study designs vary between healthy and uncomplicated malaria participants and CM participant cohorts, we have delineated the safety endpoints and time of measurements relative to the study design and risk–benefit profile for each cohort.

### Primary endpoints

In adult participants, the primary endpoint is any grade 2 or 3 AE or SAE in the 14 days after DON administration. In pediatric participants with CM, the primary endpoint includes any SAEs at any time within 14 days after DON administration. The list of solicited AEs is based on known AEs from previous DON trials. All other AEs are documented as unsolicited events. AE grading (Tables 2 and 3) was adapted from the National Institute of Allergy and Immunology (NIAID) Division of AIDS (DAIDS) toxicity tables criteria [43].

**Table 2:** Toxicity grading scales for adult participants

Parameter	Grade 1	Grade 2	Grade 3
White blood cells (10^3^/μL)	2.0 to 2.39 if normal baseline 35% reduction from baseline, if abnormal baseline	1.5 to 1.99 if normal baseline 50% reduction from baseline, if abnormal baseline	Less than 1.5 if normal baseline 65% reduction from baseline, if abnormal baseline
Hemoglobin (g/dL)			
Healthy male Normal baseline	10 to 10.9	9 to < 10	< 9
Healthy female Normal baseline	9.5 to 10.4	8.5 to < 9.5	< 8.5
Uncomplicated malaria^a^	35% reduction from baseline	50% reduction from baseline	65% reduction from baseline
Platelets (10^3^/μL)	100 to 116 if normal baseline 35% reduction from baseline, if abnormal baseline	50 to 99 if normal baseline 50% reduction from baseline, if abnormal baseline	< 50 if normal baseline65% reduction from baseline, if abnormal baseline
Serum creatinine	1.1–1.3 × ULN	> 1.3 to 1.8 × ULN	> 1.8 × ULN
Serum SGOT	1.25 to < 2.5 × ULN if normal baseline	2.5 to < 5.0 × ULN if normal baseline	≥ 5.0 × ULN if normal baseline
	35% increase from baseline, if abnormal baseline	50% increase from baseline, if abnormal baseline	65% increase from baseline, if abnormal baseline
Diarrhea	3 or more loose stools in 24 h, treated with dietary changes	6 or more loose stools in 24 h requiring IV rehydration for clinical stabilization, resolving within 36 h	≥ 6 loose stools in 24 h requiring IV rehydration for clinical stabilization, not resolving within 36 h
Gastrointestinal bleeding	Coffee ground emesis or positive stool hemoccult or frank bleeding with < 10% decrease in hematocrit/PCV	Coffee ground emesis or positive stool hemoccult or frank bleeding with a ≥ 10% decrease in hematocrit/PCV not requiring transfusion	Coffee ground emesis or positive stool hemoccult or frank bleeding with a ≥ 10% decrease in hematocrit/ PCV requiring transfusion
Mucositis	Erythema of the mucosa	Patchy pseudomembranes or ulcerations	Confluent pseudomembranes or ulcerations OR mucosal bleeding with minor trauma
Hematuria	Microscopic hematuria	Frank hematuria with normal serum creatinine (within the normal range for age)	Frank hematuria with elevated (outside of the normal range for age) serum creatinine
Nausea	Nausea with decreased oral intake not requiring intravenous rehydration	Nausea with decrease oral intake requiring intravenous rehydration	Inadequate oral fluid and caloric intake with resulting hypotension or weight loss
Vomiting	1–3 episodes in 24 h with no or mild dehydration	4–6 episodes in 24 h or any amount with moderate dehydration requiring intensive intravenous rehydration	> 6 episodes in 24 h or any amount of vomiting resulting in persistent hypotension

*ULN* Upper limit of normal, *SGOT* Serum glutamic-oxaloacetic transaminase

^a^Platelets and hemoglobin are expected to be abnormal in participants with uncomplicated malaria

**Table 3:** Toxicity grading scales for pediatric participants

Parameter	Grade 1	Grade 2	Grade 3
White blood cells (10^3^/μL)	2.0–2.39 if normal baseline	1.5–1.99 if normal baseline	< 1.5 if normal baseline
			65% reduction from baseline, if abnormal baseline
	35% reduction from baseline, if abnormal baseline	50% reduction from baseline, if abnormal baseline	
Hematocrit (PCV)	Not applicable as ongoing anemia and need for blood transfusions not unexpected		
Platelets (10^3^/μL)	100–116 if normal baseline	50–99 if normal baseline	< 50 if normal baseline
		50% reduction from baseline if abnormal baseline	65% reduction from baseline, if abnormal baseline
	35% reduction from baseline, if abnormal baseline		
Serum creatinine	35% increase from baseline	50% increase from baseline	65% increase from baseline
Serum SGOT	1.25 to < 2.5 × ULN if normal baseline	2.5 to < 5.0 × ULN if normal baseline	≥ 5.0 × ULN if normal baseline
			65% increase from baseline, if abnormal baseline
		50% increase from baseline, if abnormal baseline	
	35% increase from baseline, if abnormal baseline		
Serum glucose	Not applicable: hypoglycemia during first 72 h of admission with CM is not unexpected and is closely monitored		
Serum lactate	Not applicable: hyperlactatemia during first 72 h of admission with CM is not unexpected and is closely monitored		
Diarrhea	3 or more loose stools in 24 h, treated with dietary changes	6 or more loose stools in 24 h requiring IV rehydration for clinical stabilization, resolving within 36 h	6 or more loose stools in 24 h requiring IV rehydration for clinical stabilization, not resolving within 36 h
Gastrointestinal bleeding	Coffee ground emesis or positive stool hemoccult or frank bleeding with < 10% decrease in hematocrit/PCV	Coffee ground emesis or positive stool hemoccult or frank bleeding with a ≥ 10% decrease in hematocrit/PCV not requiring transfusion	Coffee ground emesis or positive stool hemoccult or frank bleeding with a ≥ 10% decrease in hematocrit/PCV requiring transfusion
Mucositis	Erythema of the mucosa	Patchy pseudomembranes or ulcerations	Confluent pseudomembranes or ulcerations OR mucosal bleeding with minor trauma
Hematuria	Microscopic hematuria	Frank hematuria with normal serum creatinine (within the normal range for age)	Frank hematuria with abnormally elevated (outside of the normal range for age) serum creatinine
Vomiting	1–3 episodes in 24 h with no or mild dehydration	4–6 episodes in 24 h or any amount of vomiting with moderate dehydration requiring intensive intravenous rehydration	More than 6 episodes in 24 h resulting in persistent hypotension

Laboratory parameters impacted by cerebral malaria are expected to be disordered prior to DON administration

*ULN* Upper limit of normal, *SGOT* Serum glutamic-oxaloacetic transaminase, *PCV* Packed cell volume

### Secondary endpoints

Secondary endpoints include each element of the composite primary endpoint to investigate dose-related toxicities, as well as serum DON levels used to calculate PK parameters of the drug. Additional secondary endpoints for all participants include PK parameters such as volume of distribution, maximum concentration (*C*_max_), time to maximum concentration (*T*_max_), area under the curve (AUC), clearance, elimination rate constant, and terminal half-life (*T*_½_). Non-compartmental analysis is used for the estimation of PK parameters.

### Exploratory endpoints

Exploratory endpoints in the pediatric cohort measure efficacy.

1. Brain volume score on MRI at admission and 24 h post-randomization, if MRI is available
2. Number of minutes of electrographic seizures within the first 12 h after DON administration, as measured by cEEG monitoring
3. EEG amplitude, frequency, and power analysis for samples collected pre-DON and at 3, 6, and 12 h post-DON administration
4. Cerebrospinal fluid (CSF) sampled before and 4 h after DON administration analyzed for a variety of parameters including, but not limited to, changes in metabolic profiles and immune markers
5. TCD phenotype and flow velocities measured before and 4 and 24 h after DON administration

### Participant timeline {13}

In adults, after the consent document is reviewed and signed by adult participants and eligibility is confirmed, participants remain in the research clinic for 24 h (Fig. 1A). Blood samples are drawn for pre-DON PK and baseline safety studies. Adults with uncomplicated malaria receive IV normal saline at a rate of 125 mL/h (maintenance). Oral antimalarials are administered in those with uncomplicated malaria. Each participant receives ondansetron (8 mg) 30 min before DON and 6 h after DON administration. Participants receive DON IV, at the pre–specified dose. Blood is drawn before and at 10 min, 30 min, 60 min, 90 min, and 3 h, 6 h, 12 h, and 18 h post-DON to calculate PK parameters. Twelve and 24 h, and again 7 and 14 days after DON administration, blood samples for safety are obtained. Adults have telephone or in-person safety interviews 3 months after receiving DON. Six months after receiving DON, adult participants return for a safety interview and physical examination.

Similarly, pediatric participants who fulfill enrollment criteria have pre-DON laboratory studies for safety and DON levels (Fig. 1B). Next, an IV line is placed, and participants receive IV fluids at standard maintenance rates. At enrollment, a lumbar puncture (LP) is performed both to rule out bacterial CNS co-infection and for DON mechanistic studies. IV artesunate is administered immediately upon confirmation of a CM diagnosis. Participants undergo a brain MRI (if available), TCD, and at least 30 min of EEG recording before administration of DON (or placebo). Participants remain hospitalized for a minimum of 24 h. Each pediatric participant is pre-medicated with ondansetron (0.15 mg/kg up to 5.0 mg) and receives DON IV or placebo at the pre-specified dose 30 min later. Ondansetron dosing is repeated at 8 and 16 h after DON administration. Due to the precariousness of CM and the young age of the pediatric participants, PK studies are employed through sparse sampling techniques at three of the following time points: 10 min, 30 min, 60 min, 90 min, and 3 h, 6 h, 12 h, and 18 h post-DON. A population PK analysis is used to estimate PK parameters in children. A repeat LP is performed 4 h after DON or placebo administration for metabolic studies. Participants remain hospitalized until their BCS is ≥ 3, they can drink unaided, and can sit with minimum assistance. Seven and 14 days after DON administration, blood samples for safety are obtained. At 1 and 6 months post-admission, surviving pediatric participants are examined for neurological sequelae and assigned a Glasgow Outcome Score (GOS-E) category.

Should any AEs or SAEs occur, they will be followed until resolution. During the 6-month enrollment period, should participants develop any clinical symptoms of concern, they are encouraged to return to visit study personnel for evaluation.

### Sample size {14}

A sample size of 72 pediatric participants was chosen for logistical and feasibility reasons and is based on previous experience, both with respect to historical case numbers and ward infrastructure, with enrollments in similar phase I studies at the study site. We calculated the power to show certain effects with this sample size. The calculations for power to detect safety signals are from participants who received DON in the first 2 cohorts (*N* = 14), the first 3 cohorts (*N* = 28), and the first 4 cohorts (*N* = 56). The calculations for the exploratory efficacy endpoints use sample sizes of *N* = 36 (8 placebo vs. 28 DON) for the first half (cohorts 1, 2, and 3), and *N* = 72 (16 placebo vs. 56 DON) total enrollment. From those calculations, we see that the assumed effect sizes needed to have sufficient study power to ascertain differences between dosing groups are not unreasonably large. Most outcomes are measured within 7 days of enrollment during hospitalization. Adherence with 6-month follow-up visits was 85% in previous studies. Thus, we expect a very low attrition rate of up to 15% at the 6 months post-randomization visit. The goal of the pediatric study is to assess safety and explore a dose–response association for any safety or preliminary efficacy outcomes. We did not power the study to detect small effect sizes in any of the exploratory efficacy endpoints evaluated in the pediatric participants. Nevertheless, there is sufficient sample size to detect large effect sizes in continuous exploratory endpoints between two doses and placebo for a preliminary benefit-risk assessment before the fourth cohort of pediatric enrollments or for the final analysis.

For dichotomous (i.e., present/absent) safety endpoints, we calculate exact 90% central confidence intervals for the rate per participant, so that we are 95% confident that the rate is less than the upper limit. If the true rate for participants receiving DON is equal to an anticipated SAE background rate of 20%, then with a sample size of 14 participants receiving DON, we have over 85% power to show with 95% confidence that the true rate is not greater than 54%. Repeating those same calculations with sample sizes of 28 (and 56), we have over 85% power to show with 95% confidence that the rate is not greater than 46% (37%).

### Recruitment {15}

Healthy adults are recruited from the Ndirande township through community referrals to the research clinic. Adults with uncomplicated malaria are recruited from the Ndirande Health Center and surrounding health clinics.

Children with CM are recruited from the Accident and Emergency Department and inpatient hospital units of QECH in Blantyre and surrounding district referral hospitals. Adequate participant enrollment will be achieved by continuous screening for potential participants in all participating centers.

## Assignment of interventions: allocation

### Sequence generation {16a}

Pediatric participants are randomized in a 7:2 ratio for DON: placebo within each of cohorts 1 + 2, cohort 3, or cohort 4. Participants randomized to DON in cohort 4 may additionally be split into two different dose groups of DON. Computer-generated random numbers are used.

### Concealment mechanism {16b}

Assignment of pediatric participants to their randomization arm is performed by sequentially numbered opaque sealed envelopes prepared with the allocation sequence before the trial begins. The envelopes are stored in a secure location with access limited to key study personnel.

### Implementation {16c}

The allocation sequence is generated electronically and transcribed into opaque envelopes. After a caregiver provides consent for a pediatric patient to participate in the study, study personnel open the next unopened sequentially numbered envelope to reveal the group assignment and dosing calculation sheet.

## Assignment of interventions: blinding

### Who will be blinded {17a}

Evaluators of safety and efficacy outcomes including study clinicians and nursing staff, as well as study participants and their parents/guardians, are blinded to the randomization assignment for pediatric participants.

### Procedure for unblinding if needed {17b}

The study pharmacist maintains an unblinded version of the allocation sequence in a secure location for use in unblinding or for replacement should the original assigned envelope be damaged or lost. The pharmacist does not participate in other trial activities and will not share the list with study team members involved in participant follow-up activities. If circumstances arise where unblinding is needed before the end of the study (such as the occurrence of a suspected unexpected serious adverse reaction), the ISMs, who are physicians who are not investigators for this trial, will assess the child’s clinical status. Their report is shared with the sponsor and the DSMB. The DSMB then makes a recommendation for maintaining the blinding or unblinding which is communicated to the study sponsor.

## Data collection and management

### Plans for assessment and collection of outcomes {18a}

Trial data is collected by trained study staff, including clinical, safety, and outcome measures such as history, laboratory values, and neurological testing results. Data are collected onto paper-based CRFs and scanned into an electronic database.

### Plans to promote participant retention and complete follow-up {18b}

During informed consent, participants (or their parents/ guardians) are encouraged to adhere to the study follow-up visit schedule. A verbal map to their home and phone numbers is collected. A visit calendar with all study visit dates is affixed to each participant’s health passport book—a document carried to all health care visits in Malawi—to serve as a reminder. Participants who miss visits are contacted by phone and/or a home visit. Participants will have study team contact details and can contact investigators as the needed.

### Data management {19}

The study uses DF Explore—a 21 US Code of Federal Regulations Part 11-compliant internet data entry system provided by the study data coordinating center. The data system includes password protection and internal quality checks, such as automatic range checks to identify data that appear inconsistent, incomplete, or inaccurate. Data are first collected by study personnel on paper-based data collection forms that have the patient’s unique study identifier code on each page. The CRFs are then scanned into electronic CRFs (eCRF) on DF Explore and moved up 3 levels, with data verification at each level. All hard copy, research-related documents such as hard copy CRFs are stored securely in a locked office at the Ndirande Research Clinic (adults) or PRW (children). Only the study team members have access to these documents.

Study records and reports including, but not limited to, eCRFs, source documents, ICFs, laboratory test results, and study drug disposition records will be retained for 2 years after a marketing application is approved for DON for CM or, if no application is filed or if the application is not approved for DON, until 2 years after the investigation is discontinued and the US Food and Drug Administration (FDA) has been notified. These documents will be retained for a longer period of up to 5 years, however, if required by local regulations. ICFs documenting future use will be maintained as long as the specimens exist. No records will be destroyed without the sponsor’s written consent.

### Confidentiality {27}

No study information or associated data may be released to any unauthorized third party without prior written approval of DMID and the participant. Subject confidentiality will be maintained when results are disseminated. The study monitor or other authorized representatives of the sponsor or governmental regulatory agencies may inspect all documents and records, and the clinical study site will permit access to such records.

All hard copy records are kept locked. All computer entry and networking programs are carried out with participant code numbers and with password-protected systems. All non-clinical specimens, evaluation forms, reports, and other records that leave the site are identified only by a coded number. The sites hold a Certificate of Confidentiality which states that researchers cannot be forced to release information that may identify the research subject, even by a court subpoena, in any federal, state, or local civil, criminal, administrative, legislative, or other proceedings. The researchers may use the certificate to resist any demands for information that would identify the subject, except as explained above.

### Plans for collection, laboratory evaluation and storage of biological specimens for future genetic or molecular analyses {33}

This study does not include human genetic testing. Adult participants and parents/guardians of pediatric participants decide if their residual specimens can be used for future research or destroyed at the end of the trial. This decision may be changed at any time by notifying the study team. Residual specimens are stored indefinitely at the Kamuzu University of Health Sciences molecular biology laboratories or Laboratory of Immunogenetics (LIG) within NIAID. Specimens may be shared with other investigators upon written request to this study’s principal investigator. The recipients of specimens will be informed that these specimens have an NIH Certificate of Confidentiality. The information provided to a recipient will not contain direct identifiable information.

## Statistical methods

### Statistical methods for primary and secondary outcomes {20a}

The primary analysis is the comparison of the active doses against placebo in pediatric participants. For the dichotomous safety endpoints, unadjusted pairwise analyses use 95% confidence intervals of the difference in proportions between dose groups using Wilson score criteria. For the exploratory efficacy endpoints of cEEG amplitude or frequency, MRI brain volume score, age and sex standardized mean, and diastolic flow pattern for TCD, unadjusted pairwise analyses will calculate the 95% confidence interval of the mean difference of each endpoint’s first post-treatment measurement between dose groups using a *t*-statistic.

### Interim analyses {21b}

After 50% of pediatric participants are enrolled, an interim analysis will assess the benefit-risk of the study drug to inform whether the study should continue. If a decision is made to continue, the dose–response relationship for both the primary safety endpoint and exploratory efficacy endpoints will inform which doses to study in the second half of pediatric enrollments.

For the exploratory efficacy endpoints of EEG amplitude or frequency, MRI brain volume score, and flow velocity on TCD, we will compare different dose groups using Welch’s *t*-test. We may use a linear regression dose response model to predict the effect size compared to placebo as a function of dose. For each exploratory efficacy outcome measured after DON administration, response may be modeled by dose group, or a more complicated model may be used such as a logit of area under the curve of the exposure profile, adjusting for disease severity at baseline.

### Methods for additional analyses (e.g., subgroup analyses) {20b}

Exploratory analyses will compare DON doses to each other to assess preliminary efficacy. We will explore the impact of covariates including age, sex, and other characteristics (admission BCS, MRI brain volume score, serum glucose, serum lactate, cEEG, and/or admission TCD phenotype) by multivariable regression on the exploratory outcomes. In this multivariable model, dose will be either a categorical variable with fixed doses as categories or will account for PK of exposure measurements by using the area under the curve or *C*_max_ in the logistic regression. This adjusted model will only include characteristics that predict outcomes as determined by bivariate analyses.

For MRI brain volume scoring, GOS-E scoring, and TCD phenotype, which all rely on physician scoring, we will report Cohen’s Kappa measure on inter-rater reliability for outcomes evaluated by more than one physician. For endpoints measured multiple times during follow-up, sensitivity analyses will explore variation of these measurements over time in different groups by including random effects for time of measurement in the multivariable regression models.

### Methods in analysis to adjust for protocol non-adherence and statistical methods to adjust for missing data {20c}

In both adult and pediatric studies, the primary population for analyses of all endpoints is all intent to treat (ITT) participants. Every effort will be made to minimize protocol deviations and minimize missing values. If there are deviations from protocol where treatment received differs from the treatment to which the participant is randomized, the primary analysis population will be “as-treated.” Because the primary goal is to determine dose safety, when dose randomized differs from dose received, we will analyze outcomes by the dose received and conduct sensitivity analyses on the ITT population with “as-randomized” assignment. To adjust for missing values in our analyses, we may use multiple imputation methods to measure dose effect while accounting for uncertainty arising from the missing value(s). We will generate 25 imputed datasets and fit the multivariate models accounting for model uncertainty as well as uncertainty from imputation.

### Plans to provide access to the full protocol, participant level-data and statistical codes {31c}

A copy of this protocol and protocol amendments are available on ClinicalTrials.gov. De-identified participant data will be made available upon reasonable request up to 2 years after publication of clinical trial results. Researchers may request data by providing a scientific proposal to the principal investigator. If the proposal is judged methodologically sound and if the researcher has signed a data access agreement, the requested data will be provided.

## Oversight and monitoring

### Composition of the coordinating center and trial steering committee {5d}

The study principal investigator (DGP) and local principal investigators (JM, YC), coinvestigators (BAR, MBL, NO), study physician (NN), trial coordinators (OMN, AML), study pharmacist (ND), and study data manager (NM) conduct daily trial oversight. The study oversight team meets bi-weekly to evaluate progress.

The sponsoring agency, NIAID, or its designee conducts site monitoring visits as detailed in the clinical monitoring plan at standard intervals or more frequently as directed by the study sponsor. Monitoring visits include, but are not limited to, review of regulatory files, accountability records, eCRFs, ICFs, medical and laboratory reports, and protocol and good clinical practice compliance. Site monitors have access to each participating site, study personnel, and all study documentation according to the DMID-approved site monitoring plan. Study monitors meet with site principal investigators to discuss any problems and planned actions and to document site visit findings and discussions.

### Composition of the data monitoring committee, its role and reporting structure {21a}

Safety oversight is conducted by a DSMB. The DSMB consists of at least three voting members, including a biostatistician experienced in statistical methods for clinical trials and a clinician with relevant expertise, who do not have scientific, financial, or other conflict of interest related to this study. The DSMB operates under the rules of a sponsor-approved charter. The DSMB reviews unblinded enrollment and demographic information, medical history, concomitant medications, physical assessments, clinical laboratory values, dosing compliance, and solicited and unsolicited AEs/SAEs on a regular basis and as needed during this trial. The DMID medical monitor and ISM (as deemed necessary) review SAEs in real time. As an outcome of each review meeting, the DSMB makes a recommendation on the decision to proceed with dose escalation, and to continue, modify, or terminate this trial.

### Adverse event reporting and harms {22}

AEs are collected within 14 days after DON administration. All AEs, including solicited local injection site and systemic (subjective and quantitative) reactions, are captured on the appropriate data collection forms and eCRFs. AE information includes event description, date of onset, assessment of severity using a protocol-defined grading system (Tables 2 and 3), relationship to study product and alternate etiology as assessed by a licensed study clinician, date of resolution, seriousness, and outcome. AEs are managed by the study team and referrals for further care are made on a case-by-case basis. All AEs are clinically followed through resolution.

### Frequency and plans for auditing trial conduct {23}

This trial may be audited by the study sponsor or their designee. No audits are preplanned.

### Plans for communicating important protocol amendments to relevant parties (e.g., trial participants, ethics committees) {25}

All protocol and consent amendments must be approved by regulatory authorities before implementation. If a proposed amendment includes changes that can affect participant safety or the scientific value of the trial, participants will be informed and required to sign an updated ICF. Substantial amendments will also be updated on clinicaltrials.gov. Regulatory review and approval occur at least annually throughout enrollment and follow-up but may cease if annual review is no longer required by applicable regulations.

### Dissemination plans {31a}

Results will be published in scientific journals. An electronic version of final, peer-reviewed manuscripts will be submitted to the National Library of Medicine’s PubMed Central (http://www.ncbi.nlm.nih.gov/pmc/) and will be made publicly available no later than 12 months after the official date of publication. Results from this trial will be disseminated through oral and poster presentations at national and international scientific conferences and local stakeholder meetings.

## Discussion

Malaria, in particular CM, is the most devastating parasitic disease of humankind [1]. With high rates of morbidity and mortality, it places a substantial burden on healthcare systems and populations resident in endemic areas. The main purpose of this study is to determine the safety of DON, a candidate adjunctive therapy for children with CM. Concurrent with safety studies, we will determine PK parameters and evaluate preliminary efficacy using three diagnostic tools predictive of disease outcome: brain MRI, EEG, and TCD. The proposed intervention is not resource intensive, can be readily implemented by trained medical staff, and would be easily scaled up to reduce mortality across low- and middle-income malaria-endemic countries. This is the first clinical trial to study DON for the CM indication. Our results will build on the current knowledge on CM adjunctive therapy, thereby serving as reference for further efficacy trials in similar settings.

All anticipated risks are mitigated during trial conduct. The most common risk of DON in humans is nausea and vomiting. Pre-medication with phenothiazine anti-emetics mitigated this risk in previous studies. Pre- and post-medication with intravenous ondansetron is used in the participants enrolled here because of the risk of phenothiazine-induced adverse events in children. Additional potential risks including diarrhea, gastrointestinal bleeding, thrombocytopenia, leukopenia, elevated creatinine, and elevated hepatic aspartate transaminases are expected. Participants are repeatedly monitored during the first 24 h of DON receipt and 7 and 14 days later. In previous clinical trials, SAEs, in addition to nausea and vomiting, were seen only after repeated DON administration over days to weeks. DON also has possible teratogenic effects, and so current or planned pregnancy in the 2 weeks after planned DON administration is an exclusion criterion. Additionally, participants are offered contraceptive methods. If participants become pregnant during follow-up, their pregnancy is followed until its completion.

Adult participants have no anticipated direct benefits. Their participation in this study produces a benefit to society, advancing knowledge about the potential toxicities of the DON in adults without and with malaria, before advancing to studies in a vulnerable target population, children with CM. Pediatric participants with CM may directly benefit if they are randomized to receive DON and if DON proves to reduce the mortality or morbidity risks of CM. Pediatric participants may also indirectly benefit from access to a superior nurse to patient ratio in the research unit as compared to hospitalization on the general pediatric wards.

If DON demonstrates safety in this study, and subsequent phase II and III studies establish therapeutic efficacy, it will be the first time that a therapy is proven effective for treating CM in children, a disease process with a profound public health impact across the African continent.

### Trial status

The study was initiated on 16 August 2022. Adults were enrolled under protocol version 1.7 dated 16 October 2022. The current protocol version is 2.1 dated 04 August 2023. The trial’s ClinicalTrials.gov identifier is NCT05478720. Anticipated recruitment completion is June 2026.

## Data Availability

The final trial dataset will be made available upon reasonable written request to the corresponding authors.

## Abbreviations

AE: Adverse event/adverse experience
AUC: Area under the curve
BBB: Blood brain barrier
BCS: Blantyre coma score
cEEG: Continuous electroencephalography
CM: Cerebral malaria
*C*_max_: Time to maximum concentration
CRF: Case report form
DAIDs: NIAID Division of AIDS
DMID: Division of Microbiology and Infectious Diseases, NIAID
DON: 6-Diazo-5-oxo-L-norleucine
DSMB: Data and Safety Monitoring Board
eCRF: Electronic case report form
ECM: Experimental cerebral malaria
ICF: Informed consent form
IRB: Institutional review board
iRBC: Infected red blood cell
ISM: Independent safety monitor
ITT: Intention to treat
IV: Intravenous
LP: Lumbar puncture
MP: Malaria parasitemia by microscopy
MRI: Magnetic resonance imaging
*N*: Number (typically refers to subjects)
NIAID: National Institute of Allergy and Infectious Diseases, NIH
NIH: National Institutes of Health
PCV: Packed-cell volume
PK: Pharmacokinetics
QECH: Queen Elizabeth Central Hospital
SAE: Serious adverse event/serious adverse experience
SGOT: Serum glutamic-oxaloacetic transaminase
TCD: Transcranial Doppler
*T*_1/2_: Half life
*T*_max_: Time to maximum concentration
ULN: Upper limit of normal
US: United States
WHO: World Health Organization
